# Temperature Impact in LoRaWAN—A Case Study in Northern Sweden

**DOI:** 10.3390/s19204414

**Published:** 2019-10-12

**Authors:** Níbia Souza Bezerra, Christer Åhlund, Saguna Saguna, Vicente A. de Sousa

**Affiliations:** 1 Department of Computer Science, Electrical and Space Engineering, Luleå University of Technology, 93187 Skellefteå, Sweden; christer.ahlund@ltu.se (C.Å.); saguna.saguna@ltu.se (S.S.); 2 Communication Engineering Department (DCO), Federal University of Rio Grande do Norte (UFRN), 59078-970 Natal, Rio Grande do Norte, Brazil; vicente.sousa@ufrn.edu.br

**Keywords:** ADR, IoT, LoRa, LoRaWAN, propagation model, smart city

## Abstract

LoRaWAN has become popular as an IoT enabler. The low cost, ease of installation and the capacity of fine-tuning the parameters make this network a suitable candidate for the deployment of smart cities. In northern Sweden, in the smart region of Skellefteå, we have deployed a LoRaWAN to enable IoT applications to assist the lives of citizens. As Skellefteå has a subarctic climate, we investigate how the extreme changes in the weather happening during a year affect a real LoRaWAN deployment in terms of SNR, RSSI and the use of SF when ADR is enabled. Additionally, we evaluate two propagation models (Okumura-Hata and ITM) and verify if any of those models fit the measurements obtained from our real-life network. Our results regarding the weather impact show that cold weather improves the SNR while warm weather makes the sensors select lower SFs, to minimize the time-on-air. Regarding the tested propagation models, Okumura-Hata has the best fit to our data, while ITM tends to overestimate the RSSI values.

## 1. Introduction

LPWA technologies have been used as IoT enablers because of their battery duration and extended coverage area. LoRa [1] is an outstanding candidate which is gaining attention nowadays, especially due to its PHY layer based on CSS [2]. It adds resistance to interference and multipath while providing flexible configuration of both bit rate and coverage range by the tuning of internal parameters, for example, the SF.

While LoRa is a PHY layer specification, LoRaWAN [3] is the MAC protocol defined to be used on top of the aforementioned PHY. It is typically developed in a star-of-stars topology where gateways are the entities responsible for forwarding the messages from the sensors to the network server.

For the suitable deployment of a LoRaWAN, the channel conditions should be taken into consideration. The location of gateways and sensors should be chosen carefully, as the signal can be corrupted by obstacles. The weather might also influence the communication between gateway and sensors, for example, when there is the presence of fog or snow, or even when the thermal amplitude is high.

In the smart region of Skellefteå, northern Sweden, Luleå University of Technology deployed a LoRaWAN for research purposes. In this region, different types of IoT services are being developed to improve the quality of life of its citizens [4]. Examples of those applications are sensors used to monitor the public illumination lights, sensors for monitoring the level of trash in the trash bins, as well as pollution sensors monitoring the air in some areas of the city. As the aforementioned sensors are widely spread across this smart region, LoRaWAN is the best choice for the overall connection infrastructure.

This paper presents investigations of a real LoRaWAN deployment under different temperature conditions. First, we provide an evaluation in terms of RSSI, SNR and SF usage when sensors are configured for ADR. Our goal is to observe the influence of the extreme changes in the weather along the year in the city of Skellefteå to the LoRa signal. Then, we propose to analyze the fitting of real-life RSSI using a RF planning tool. Our target is to present a comparative graphical evaluation for the Okumura-Hata [5] and the ITM [6] propagation models. Therefore, to the best of authors’ knowledge, there is no vast literature addressing the evaluation of temperature conditions into a LoRaWAN real-life deployment, especially with the amount of data provided in our experiments. In this paper, our key contributions are:

- Evaluation of RSSI and SNR values from our LoRaWAN deployment regarding different weather conditions, over the course of eight months;
- Comparison of real-life RSSI values collected from the network to values generated using an RF planning tool (CloudRF [7]) with two different propagation models;
- Evaluation of the ADR in terms of SF distributions in our LoRaWAN deployment (under different weather conditions and over the course of eight months).

This paper is organized as follows—Section 2 discusses the literature regarding LoRa and LoRaWAN, with focus on propagation models and the impact of environmental factors in LoRa performance. Section 3 introduces LoRa and LoRaWAN technologies, while Section 4 presents the LoRaWAN deployment and the corresponding equipment used during our experiments. The radio planning tool and evaluated propagation models are presented in Section 5. Section 6 defines the measurement scenarios and the collected metrics. The results are presented and discussed in Section 7. Section 8 concludes the paper and presents a summary of the main results and future work.

## 2. Related Work

LoRaWAN has proven to be a topic of interest for many researchers. The authors of Reference [8] present a survey about LoRaWAN, while in References [9,10,11] LoRaWAN is used for IoT applications in smart city scenarios. Improvements in LoRaWAN design and some of its features (like ADR) are presented in References [12,13] and the authors of Reference [14] present a performance analysis of offshore transmissions done using a LoRaWAN.

In Reference [15], the authors evaluate their own-built LoRa, called LoRa FABIAN using parameters such as packet error rate, RSSI and SNR. Results show evidence of a meaningful coverage enhancement with the elevation of the gateway’s antenna. Although providing discussions about the influence of the environment into the quality of device-gateway communication, they do not use LoRaWAN as the MAC protocol. Analytical and simulation studies are presented in Reference [16] for a single gateway LoRa network. Uplink outage probability (the probability that a packet will not be transmitted properly) is formulated and the impact of low SNR and co-spreading sequence is evaluated. The model proposed is validated only using simulation and the model is only valid for the single gateway scenario. The performance of a real LoRaWAN deployment in the city of Oulu, in Finland, is presented in Reference [17]. The network provides connectivity for 15 km on the ground and over 30 km on the water. They also proposed a channel attenuation model with the collected measurements but the temperature is not included in their model.

The comparison of results obtained by a radio planning tool and measurements from a real LoRaWAN deployment is shown in References [18,19,20]. The authors of Reference [18] estimate the coverage range using the Okumura-Hata model for three different scenarios—urban, suburban and rural. They then perform a drive-test with a sensor on the top of a car collecting RSSI and packet delivery measurements. Different DR and CR are evaluated and the best configuration observed is using the lowest DR and the highest CR. Their estimation from the radio planning tool is closer to the coverage range seen in the experimental measurements. The authors of Reference [19] compare real-life LoRaWAN data collected in the city of Glasgow to a LoRaWAN data generated using the ns-3 open-source simulator for three propagation models—Okumura-Hata, COST-231 Hata and COST-231 Walfish-Ikegami. The Okumura-Hata propagation model has the best fit to the measured data.

In our previous work [20], we perform a comparison of the RSSI values from our LoRaWAN deployment in Skellefteå to values calculated using CloudRF tool for a short period of time. We use three propagation models—ITM, ITWOM [21,22] and Okumura-Hata, with the ITWOM providing the best fitting. Although in Reference [20] we use three propagation models, for this work we used only two. Due to a recent upgrade, CloudRF engine has discontinued some functionalities regarding ITWOM model, for example, we cannot calculate an RSSI value at a specific location (BSF). Despite those results from real measurements and curve-fitting by commercial planning tools and open-source simulators, the authors do not evaluate how changes in temperature affect the RSSI and SNR, as well as the SF allocation when ADR is enabled.

Evaluation of the temperature impact in LoRa/LoRaWAN is limited in literature but there are recent contributions [23,24,25]. In Reference [23], the authors present an experimental study on LoRa reliability in outdoor and indoor scenarios, showing LoRa sensitivity to temperature and humidity. They use a controlled laboratory environment, where the LoRa nodes were heated using a light bulb, under temperatures varying from 0 to 60 °C. They established temperatures which make LoRa communication unreliable. While the evaluation performed in Reference [23] focused on the temperature of the LoRa sensors, we focus on the environment temperature, including the whole system (sensor, gateway and the communication channel). The LoRa channel characterization in the Antarctic is presented in Reference [24]. The authors investigate both the 434 MHz and the 868 MHz carriers in terms of coverage and RSSI. LoRa communication in the Antarctic is possible at a distance of 20 km, as long as it is in a LoS condition. NLoS communication is also possible but with significant packet loss. Different from our work, the experiment lasted a single day and they use directional antennas in both receiver and transmitter. The authors of References [25,26] perform experimental studies on the impact of temperature on the LoRa performance. They confirm the disruption of the communication link with the temperature increasing, making the packets exchange impossible. They also investigate how different PHY settings can be affected by fluctuating temperatures. However, an accurate selection of LoRa PHY parameters can considerably counteract the negative effects of high temperatures. Reference [26] especially evaluates how vegetation and different antennas influence LoRa communication. Their approaches are different from the herein proposed because they are more interested in the impact of higher temperatures in LoRa, in non-urban environments and without evaluating LoRaWAN features like ADR.

## 3. LoRa and LoRaWAN

LoRa is the PHY layer specification defined to be used with LoRaWAN MAC protocol [27]. Since its creation by Cycleo, a company bought by Semtech [28] (nowadays, the technology onwer), LoRa has become a promising technology for the IoT because of the CSS modulation [2].

CSS was developed in the 1940s, especially for military applications. However, its low transmit power requirements and propagation robustness have made CSS a popular choice among different communications applications in recent years. In LoRa, a continuously frequency varying chirp signal is what enables the spread of the spectrum [29], providing LoRa robustness against Doppler, multipath and interference effects [30].

A flexible configuration is one of the advantages of LoRa. It is possible to increase/decrease the bit rate while decreasing/increasing the range, by the simple modification of the following parameters—BW, SF and CR. In the EU863-870 ISM band, the possible values for BW are 125 kHz, 250 kHz and 500 kHz, while the SF can vary from 7 to 12. The CR improves the reception robustness by encoding 4-bit data with redundancies for error correction, assuming values of 4/5, 4/6, 4/7, or 4/8. Thus, the LoRa bit rate, in bits per seconds, is calculated as follows:

(1) $$R_{b}=SF\cdot \frac{1}{\left[\frac{2^{SF}}{BW}\right]}$$

According to Equation (1), SF is a dominant factor for the bit rate calculation, defining the transmission PHY Data Rate (DR), as illustrated in Table 1 [31]. The table omits the DRs from 6 to 14 because they are not implemented in commercial LoRa for the carriers in the EU863-870 ISM band.

Another important feature of LoRa is the possibility of the network server configuring the SF of devices according to the propagation conditions. This feature is called Adaptive Data Rate (ADR) and it allows the sensors to use the RF resources in a more efficient way. The ADR procedure can be performed by the network server or by the device [3]. If the propagation conditions change rapidly and frequently, then the procedure is performed by the device. Otherwise, the network server is responsible for the process.

When the network server starts the ADR, it changes both the SF and the transmission power of a device, according to Reference [31]. However, this is a vendor-specific implementation. Thus, most of the commercial implementation of network server managed ADR are trade secrets and it is vendor dependent. In general terms, the process starts with the collection of SNRs samples from messages sent by the devices through the gateways. Then, the network server, based on the highest SNR collected in any of the gateways within the sensors range, sets a specific SF, which better reflects the channel conditions. The SF is defined according to the following rules [31]—(i) if the channel condition is poor (i.e., low SNR), a higher SF is set; and (ii) if the channel condition is good (high SNR), a lower SF is set. The transmission power is quantized into eight levels with 16 dBm of maximum transmission power (level 0) and the following levels with 2 dBm less power [31]. According to Reference [3], the transmission power level should be defined to increase the battery life of the end-device and maximize the network capacity.

On the other hand, if the device initiates the ADR process, it changes only the SF, also based on the SNR values but in a way to minimize the total time on air. In practical terms, the device managed ADR process tends to select low SFs, as they are the ones with the lowest time on air.

**Figure 1:**
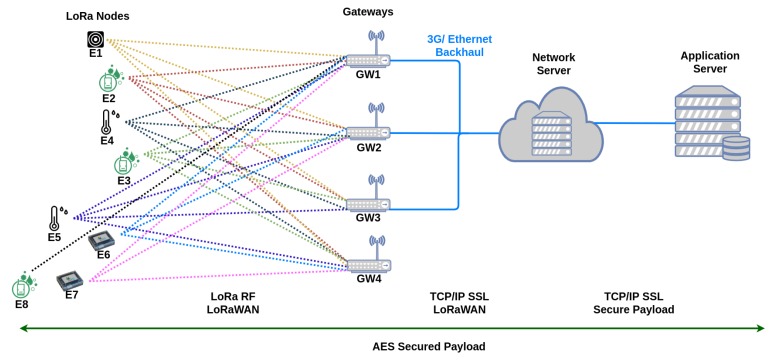
Illustration of the LoRaWAN topology based on Reference [32]. The Application Server is not used in our experiments.

As previously stated, LoRaWAN, a LPWA networking protocol for LoRa PHY layer, is composed of three main components—the LoRa nodes, the gateways and a network server. A LoRaWAN is normally deployed in a star-of-stars topology, with the nodes transmitting data to the gateways, which act as routers, forwarding the packets from the nodes to the network server. The nodes are not connected to a specific gateway. Instead, they are connected to a specific network server and any gateway in the range of a node can receive its packets and forward them to the network server. Figure 1 presents the LoRaWAN topology based in the topology described in Reference [32]. The dotted lines represent wireless connections, while the continuous lines represent cabled connections (although, a wireless connection between the gateways and the network server is also possible). The green line below the network nodes indicates the communication protocol used by them to exchange messages in the network. It is worth mentioning that for our experiments we do not use any Application server.

There are three different device classes defined in the LoRaWAN standard [3]—Classes A, B and C. Class A devices transmit in an ALOHA-type protocol, where two short receiving windows follow every uplink transmission. Devices implementing this class are the most energy-efficient ones and all LoRaWAN devices must implement at least this class functionality. Class B devices allow for more receiving windows, in contrast to Class A. Devices implementing this class can, when scheduled, open extra receiving windows for downlink traffic. The gateway sends a time-synchronized beacon to the node informing when the receiving window should be open. Class C devices only close their receiving windows when transmitting. From the three classes, this is the one that is the least energy-efficient (as it is constantly “listening” to the channel).

## 4. Experimental Setup

Our LoRaWAN is composed of four Kerlink Wirnet iBTS Compact outdoor gateways [33] and one network server. The gateways can be remotely monitored and managed with Kerlink network operations solution. We use the software version 2.3.3. The network server is the Wanesy Management Center [34], also from Kerlink. It is Kerlink’s network operations solution, where all the information about the equipment, configuration, received/sent messages can be accessed. The gateways have vertically polarized omnidirectional antennas with a maximum gain of 3 dBi, configured for transmission/reception in 8 channels with 125 kHz of bandwidth. The LoRaWAN in Skellefteå follows the 1.0.2 specification [3] and operates in the 868 MHz frequency carrier. Table 2 gives the location of the employed gateways.

We used eight sensors positioned at a different location around the city. Five of those sensors (E1 to E5) are Elsys LoRa^®^ ELT-1 [35] stationary sensors (commercial LoRaWAN devices from Elsys). Each sensor has an omnidirectional antenna with a maximum gain of 3 dBi. They are battery-powered and mounted along the oldest wooden bridge in Sweden (Lejonström bridge) at approximately 2 m above the ground level. The sensor E8 is also Elsys, model ERS [36], for indoor environments. The other two devices (E6 and E7) are mcf88 devices, model MCF-LW06485 [37], (which is an interface to a wide range of sensors used mainly in industrial applications). They are mounted on lampposts and both are powered by the lamppost to which they are connected, which means that they are only powered up when the lamppost is lit. The device E6 is mounted at ground level while the E7 one is located at 1 m above the ground level. Table 3 presents the list of sensors with their respective configuration and location.

According to Table 3, sensors E2 and E4 are located at the same position, as well as E3 and E5. With this combination, we could analyze the influence of the pair position-SF on the RSSI and SNR.

The sensors send data to the gateways within their coverage area and the gateways forward the data to the network server. The Wanesy Management Center (our network server) implements a REST API [38], which we use to get data from the network server. We parse and plot data with Python and Matlab scripts, respectively.

## 5. Propagation Models and Radio Planning Tool

Propagation models are used to describe the propagation characteristics of a wireless signal in regards to the environment, physical effects of radio wave and the frequency. Several propagation models have been described in the literature since the 1940s [39]. For this work, we compare data collected from our LoRaWAN network with estimation of two propagation models available within CloudRF commercial tool [7]. The propagation models selected are Okumura-Hata [5] and ITM [6], and are briefly described in the following sections.

### 5.1. Okumura-Hata

The Okumura-Hata is a combination of the equations from the Hata model, used to fit the curves from the Okumura model [5]. Such curves come from measurements taken by Okumura in the city of Tokyo, Japan, in 1968. It is an empirical model with parameters that account for the type of the environment, carrier frequency, the base station/gateway and antenna height of a device and the transmitter-receiver distance in kilometers. It is suitable for frequencies between 150 MHz and 1500 MHz, base station/gateway antenna height from 30 m to 200 m and antenna height of devices from 1 m to 10 m. It has equations for the following environments—rural, suburban, urban and metropolitan areas. One disadvantage of the Okumura-Hata model is that it does not consider the terrain profile in its calculation. We chose this model for being the most extensively used for wireless communications applications and for this reason, we refer the reader to Reference [5] for a complete description of this model’s equations.

### 5.2. ITM

The ITM model, also known as the Longley-Rice model, is a model created by the NTIA agency to plan the broadcast deployment of VHF transmitters in the US at the end of the 1960s. It is used for broadcast coverage for frequencies between 20 MHz and 20 GHz and generally used for point-to-point communications. It is defined as a two-part system containing in the first part the ITM core and in the second part, an input-output package. It has two operation modes, the area prediction mode and the point-to-point mode and they differ by the amount of input data necessary to the model calculation. In the point-to-point mode it uses terrain data to calculate the path loss. However, this model is based on the classical diffraction theory, which does not provide precise calculations of radio waves over irregular terrain. Also, it does not provide corrections due to environmental factors in areas closer to the receiver. As this model is defined as a two-part system, we refer the reader to Reference [6] where detailed information about how the two parts are implemented can be obtained. The ITM model was selected in this study because it is the model recommended by the RF planning tool for LoRa applications.

### 5.3. Radio Planning Tool

CloudRF [7] is the tool we used to estimate the coverage area for the four LoRa gateways we have in place, as well as to calculate the RSSI for each one of our sensors in regards to each gateway. It allows us to configure the receiver and the transmitter, creating a simulated environment that considers some characteristics of the real equipment. It also allows us to configure the propagation model with the help of topographic maps to better estimate the terrain impact on the signal propagation. We show the configuration parameters of CloudRF in Table 4 and Table 5.

**Figure 2:**
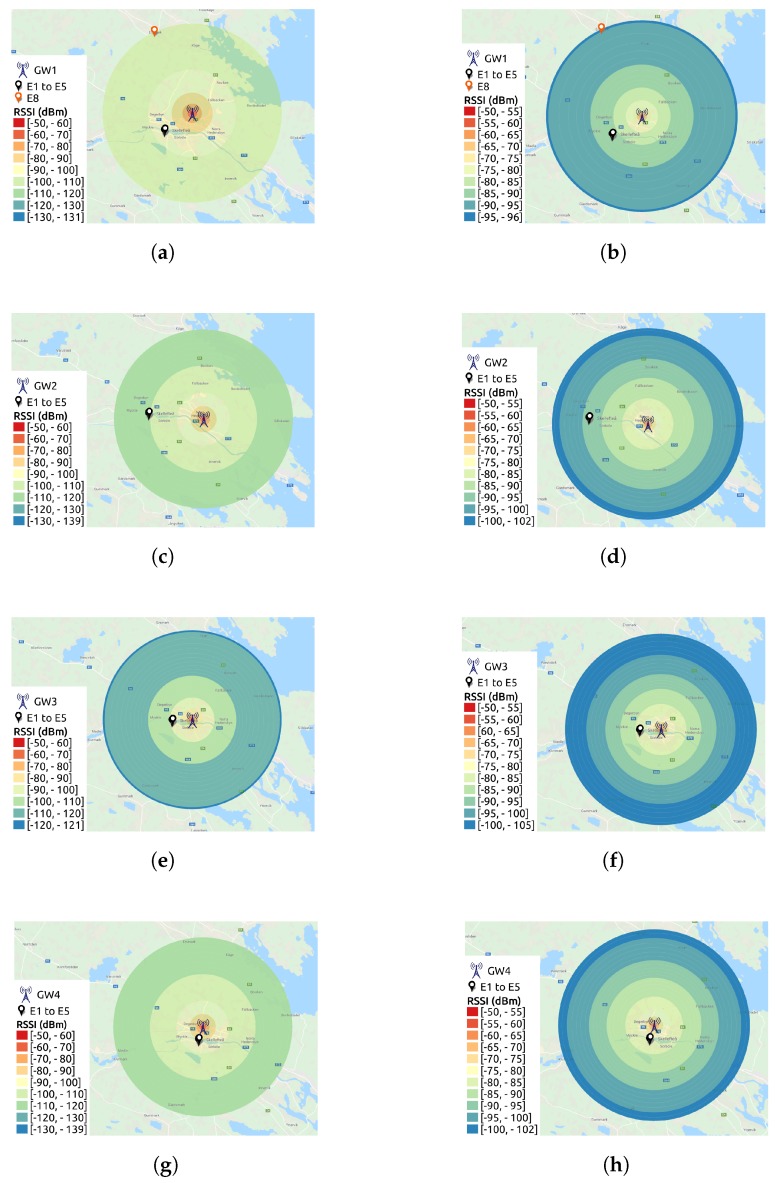
RSSI coverage graphs by CloudRF [7], where the city of Skellefteå is in the center of the coverage circles. The map layer is from Google Maps [41]. (**a**) GW1—Okumura-Hata. (**b**) GW1—ITM. (**c**) GW2—Okumura-Hata. (**d**) GW2—ITM. (**e**) GW3—Okumura-Hata. (**f**) GW3—ITM. (**g**) GW4—Okumura-Hata. (**h**) GW4—ITM.

Using the parameters shown in Table 4 and Table 5, for every gateway, we have a coverage graph showing the estimated RSSI interval for each propagation model tested. Those graphs are presented in Figure 2.

In order to calculate the RSSI for each sensor, we first need to create a network of two or more gateways. We add every gateway with their characteristics, saving all of them in a single network. We then use the Best Server Feature (BSF) of CloudRF [40]. This feature allows us to calculate the RSSI at a specific point in the map (we used the location of our sensors), based on the previously created network. Once we select the network and add the latitude, longitude and altitude information from our sensor, we have the computation of the RSSI for all gateways in the network. Table 6 shows the results we obtained with this feature.

The RSSI ranges from each coverage graph presented in Figure 2 are used in Section 7 for comparison with the RSSI values collected from our LoRaWAN deployment. It is worth mentioning that all the maps presented in this paper were created using data from Google Maps [41]. A preliminary analysis of Figure 2 shows Okumura-Hata as more conservative (providing lower RSSI values) than the ITM model.

## 6. Measurement Scenarios and Evaluated Metrics

We performed measurements over the course of eight months (from September 2018 to May 2019). Thus, we evaluated the effect of temperature changes in the signal propagation (RSSI and SNR performance) covering a broad range of temperature variations, starting from the autumn, passing through a rough winter and a very short spring, to finally consider higher temperatures in the early summer. Skellefteå is a city with subarctic climate, with temperatures reaching −30 °C in the winter and up to 33 °C on the warmest summer day. Table 7 presents the periods, the average temperature, as well as the minimum and maximum temperatures for each period, we performed our experiments. The temperatures were collected from [42], which is a weather station on top of a high school building in the center of Skellefteå. The table shows results from the coldest period to the warmest. Periods are labeled from I1 to I5 for simplicity when explaining the results. Table 7 also shows information about equipment. Some of our gateways were in maintenance during specific periods and some sensors run out of battery after I1 or were mounted at their positions after I4 and/or I3.

We organized our results in four scenarios as described below:

- Scenario 1 (S1): Gateway-sensor LoS link, outdoor sensors with fixed SF (sensors E1 to E5) and gateways GW1, GW2 and GW4;
- Scenario 2 (S2): Gateway-sensor LoS link, indoor sensor with fixed SF (sensor E8) and gateways GW1, GW2 and GW4;
- Scenario 3 (S3): Gateway-sensor LoS link, outdoor sensors with ADR enabled (sensors E6 and E7) and gateways GW1, GW2 and GW4;
- Scenario 4 (S4): Gateway-sensor NLoS link, outdoor sensors with fixed SF (sensors E1 to E5) and gateway GW3.

We compare data from our LoRaWAN deployment to the data generated using CloudRF for scenarios S1, S2 and S4 (the ones without ADR). Each of these scenarios and the metrics collected in our experiments are described in the following sections.

### 6.1. Scenario 1 (S1)—Outdoor with Fixed SF

**Figure 3:**
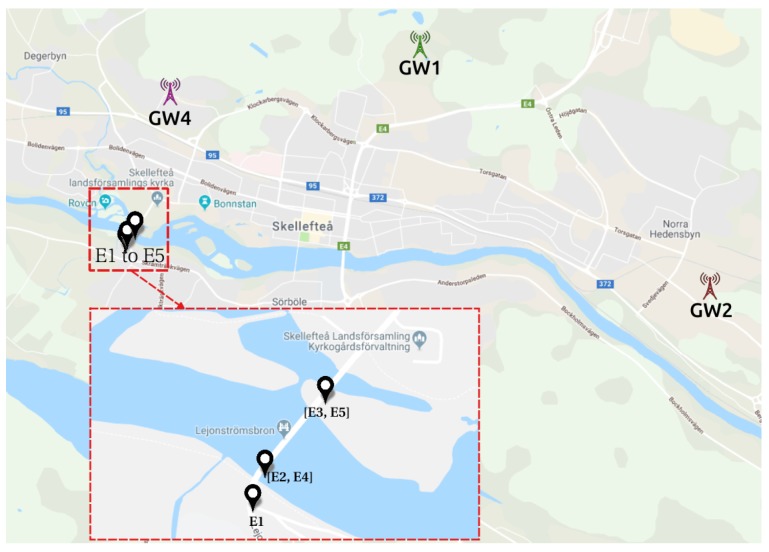
S1—gateways and sensors positioning. Map layer from Google Maps [41].

In this scenario, referred henceforth as S1 for the sake of simplicity, we positioned five sensors (E1 to E5, described in Table 3) along a wooden bridge. Each sensor has a specific SF and ADR is disabled both in the node and in the network server. Figure 3 shows the map with the location of the sensors and the gateways.

**Figure 4:**
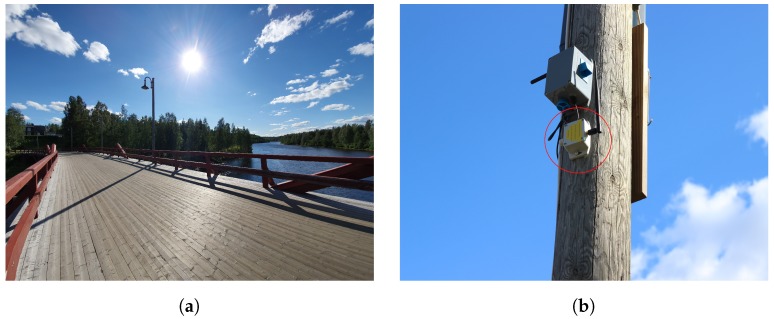
Wooden bridge and E1 sensor close-up. (**a**) Wooden bridge (Lejonströmsbron). (**b**) Close-up of E1 sensor.

The bridge is above the Skellefte river. It is a 173 m long bridge for both vehicles and pedestrians, where we positioned the sensors on the lampposts of the bridge. Figure 4 shows the bridge and a close-up of the E1 sensor (the one inside the red circle).

For this scenario, we evaluate how the city landscape, as well as the different temperatures along the year interfere in the signal propagation conditions. Our results cover the RSSI and SNR metrics for all periods described in Table 7.

### 6.2. Scenario 2 (S2)—Indoor with Fixed SF

**Figure 5:**
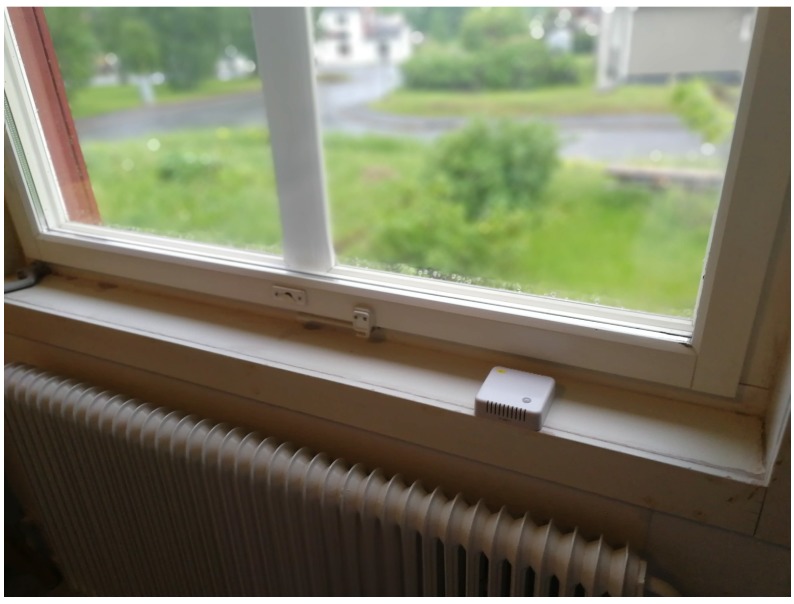
Sensor E8 beside a 2 layer glass window inside of a wooden house.

E8 is the sensor used in this scenario (see its configuration in Table 3). It is located inside a wooden house at approximately 1 m from the ground level beside a two-layer glass window. The window is facing the south-west direction. Figure 5 shows the sensor positioning inside the house.

**Figure 6:**
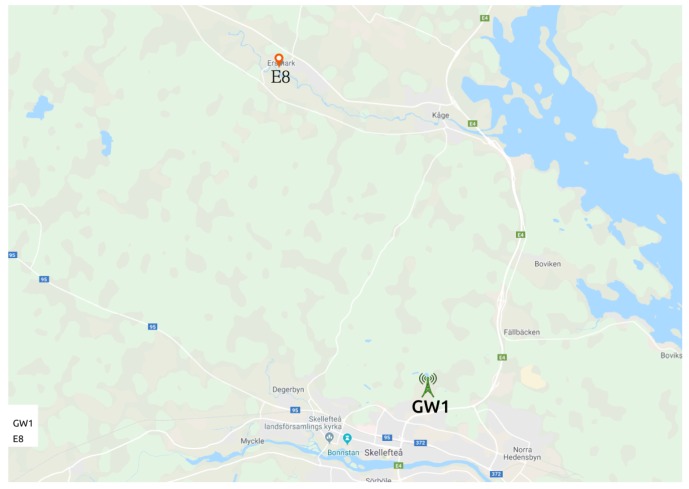
S2—gateway and sensor positioning. Map layer from Google Maps [41].

We positioned the sensor at approximately 10 km in a straight line from the only gateway it reaches (GW1). Figure 6 presents the map showing E8 and GW1.

As E8 has been operating since 20 October 2018, we have its measurements during I1, I2, I3 and I5 periods. The ADR mechanism is also disabled in this scenario.

### 6.3. Scenario 3 (S3)—Outdoor with ADR Enabled

Devices (E6 and E7) in this scenario are configured with ADR enabled, which means their SF changes according to the channel conditions.

**Figure 7:**
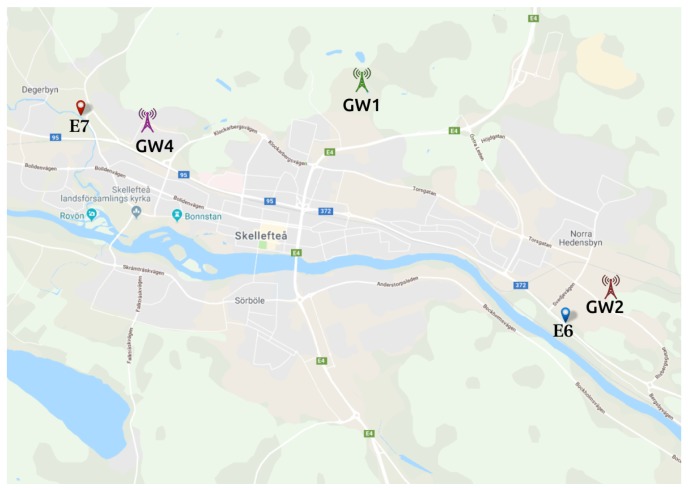
S3—gateways and sensors positioning. Map layer from Google Maps [41].

As presented in Table 7, E6 and E7 are active only during periods I1, I2 and I5, because we connected them to our LoRaWAN on 20 December 2018. Figure 7 shows the gateways and the sensors locations for S3.

### 6.4. Scenario 4 (S4)—Gateway—Sensor NLoS Link

**Figure 8:**
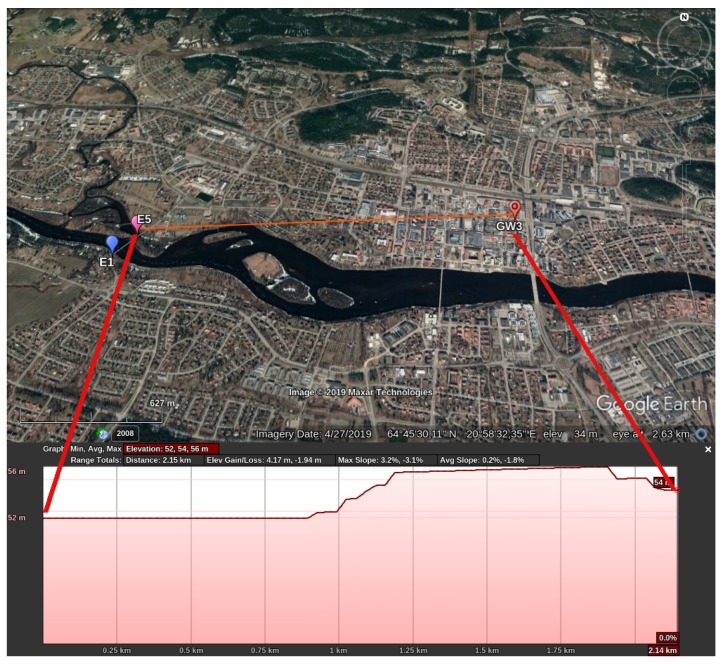
Elevation profile for E5 in relation to GW3 created with Google Earth [45].

Scenario S4 is conceived due to a particularity of GW3—it is the gateway which has an NLoS link to the sensors. Figure 8 shows the elevation profile between E5 and GW3 but the profile is the same for E1. Differently from the aforementioned scenarios, GW3 is located at the center of Skellefteå, on top of a building surrounded by other buildings of the same height. In an NLoS link, the city landscape plays an important role in the radio propagation, once the signal relies on multipath propagation to arrive at the receiver. Additionally, Skellefteå is experiencing a burst of new constructions, with two significant structural changes happening around GW3 position—(i) the House of Culture [43], whose construction began on December 10, 2018; and (ii) apartment buildings and a garage building [44] (right next to each other), whose construction began on 3 September 2018. Those constructions might influence the link between GW3 and the sensors.

GW3 is also affected by the fluctuations in temperature caused by the surrounding buildings during fall and winter, as well as the heated street nearby the building where it is located (Nygatan). In Sweden, the majority of buildings and houses use district heating [46], where the heating is generated at a central boiler station and distributed through a network of pipes. Those pipes are very well insulated, so that the heat does not escape while traveling to the buildings/houses. For the street which passes in front of the building where GW3 is deployed, the insulation was removed to allow for the heating of such street. Buildings and houses are also well insulated, to keep the warm air inside. However, they have ventilation systems, which expels part of the warm air to maintain the air quality indoors.

**Figure 9:**
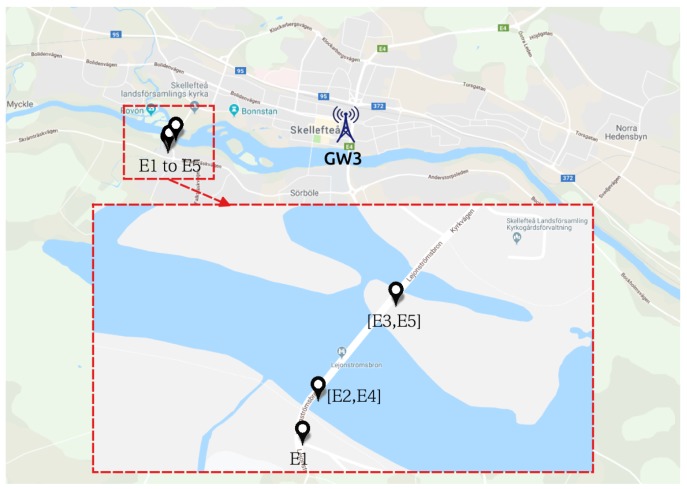
S4—gateway and sensors positioning. Map layer from Google Maps [41].

During the coldest periods of the year, like I1 and I2, the heating system in all buildings in Skellefteå is turned on and the ventilation will be expelling part of the warm air. This causes an increase in the temperature around GW3, as the surrounding buildings are expelling warm air to the outside. Figure 9 shows GW3 position in relation to the sensors.

## 7. Results Presentation and Discussion

This section presents and discusses the results for each scenario previously mentioned.

For S1 and S2, we compare the data from our LoRaWAN to the data generated using CloudRF. We compare the RSSIs CDF per period to the values generated with the Best Server Feature from CloudRF, as well as to the range values of the RSSI estimation maps. We also observe the SNR CDFs to evaluate the impact of the temperature variations along the year to the LoRa link. We drawn separate plots for every gateway.

We observe the ADR behavior in scenario S3. Thus, our experiments focus on SF usage. We evaluate the impact of the weather conditions in the choice of SF by presenting SF histogram per period, combined in a single plot, one for every gateway.

Scenario S4 presents results regarding the NLoS link between gateway GW3 and the sensors, following the same criteria employed in scenarios S1 and S2.

Table 8 summarizes all scenarios configuration, including their corresponding assessment targets.

### 7.1. S1 Results

For this scenario, we present results for only two sensors out of five, using different SFs, named E1 and E5. This is done for brevity and because we observe similar results for sensors that are less than 50 m apart from each other.

While for the E5 sensor we have data for all periods (from I1 to I5, according to Table 7), for E1 we only have data from I1 to I4. This happens because E1’s battery had run out on 24 January 2019; thus, the sensor did not make any transmissions after this day.

Our first target is to compare the RSSI values generated by CloudRF to our measured RSSI data for E1 sensor and different gateways. We plot RSSI CDFs for all periods in a single plot per gateway. The vertical lines plotted together with the CDFs correspond to the values calculated by the Best Server Feature from CloudRF. For simplicity, they are named in the figures according to the legends in Table 6 (ITM for the ITM model and O-H for the Okumura-Hata model). Additionally, the plots have two rectangles to represent the RSSI range extracted from the CloudRF maps in Figure 2. The orange rectangle shows the range for the ITM model, while the purple rectangle shows the range calculated for the Okumura-Hata model.

**Figure 10:**
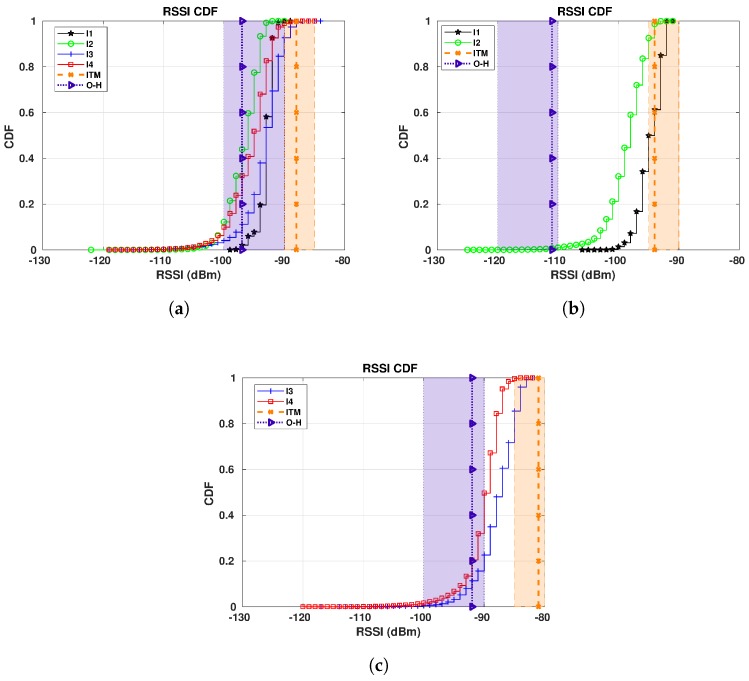
E1 RSSI CDFs for all gateways: I1 (16/01/2019 to 07/02/2019): −16.96 °C on average; I2 (22/12/2018 to 06/01/2019): −9.45 °C on average; I3 (25/10/2018 to 04/11/20018): −0.15 °C on average; I4 (30/09/2018 to 14/10/2018): 6.56 °C on average. (**a**) E1 RSSI CDFs for GW1. (**b**) E1 RSSI CDFs for GW2. (**c**) E1 RSSI CDFs for GW4.

Figure 10 shows the RSSI CDFs for sensor E1. Starting from GW1, Figure 10a and analyzing the Okumura-Hata estimations, the RSSI estimated values for the area where the sensor is located lies between −90 and −100 dBm, according to Figure 2a. This estimated RSSI range covers the RSSI values starting from the 10th-percentile and up to the 90th-percentile for all the periods of measured data. Then, the vertical line representing the value calculated with the Best Server Feature for this sensor to this gateway (−97 dBm) crosses all the CDF curves, meaning that this value is found in every period. Thus, the Okumura-Hata model fits our data for this sensor and this gateway. Looking at the ITM model for the same gateway, Figure 2b, the estimated RSSI values are between −85 and −90 dBm for E1’s location. Since only approximately 16% of the results for I3 (25/10/2018–04/11/2018), that is, from the 84th to the 100th-percentile in the corresponding CDF are within the calculated range, the ITM model overestimates the RSSI.

We reach a different conclusion for GW2 (Figure 10b). The Okumura-Hata estimation (−110 to −120 dBm), Figure 2c, is only inside the very beginning of the I2 (22/12/2018–06/01/2019) curve, while nothing of the I1 (16/01/2019–07/02/2019) curve is within the Okumura-Hata interval. In this case, only approximately 0.5% of the I2 data is within the interval calculated by CloudRF. However, for the ITM model, Figure 2d, the RSSI estimation (−95 and −90 dBm) covers from the 35th-percentile to the 100th-percentile for I1 and from the 84th-percentile to the 100th-percentile for I2. Thus, for this gateway, ITM model presents a better fit than Okumura-Hata.

The Okumura-Hata model is also the best model for GW4. Its estimation (−90 and −100 dBm) covers from zero to the 23rd-percentile for I3 (25/10/2018–04/11/2018) and from zero to the 50th-percentile for I4 (30/09/2018–14/10/2018), while the estimation of ITM model, Figure 10c, covers only approximately 28% of the data for I3 (from the 72nd to the 100th-percentile).

Summarizing the conclusions, our first investigation reveals an overestimation of LoRa RSSI by the ITM model, with the Okumura-Hata providing better fitting to the measured data. However, as expected, the gateway location also influences the propagation conditions and, as a consequence, the choice of the best propagation model. Our results also show a clear impact of weather condition to LoRa RSSI behavior. Thus, there is indeed a need to improve propagation models to include the weather seasonality, especially for estimation in regions such as Skellefteå, where the thermal amplitude is high.

**Figure 11:**
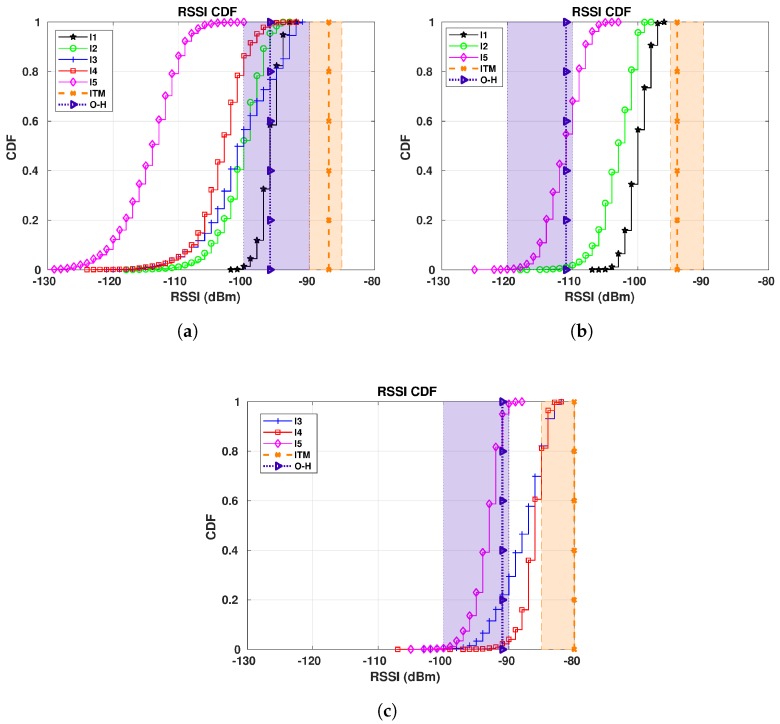
E5 RSSI CDFs for all gateways in S1: I1 (16/01/2019 to 07/02/2019): −16.96 °C on average; I2 (22/12/2018 to 06/01/2019): −9.45 °C on average; I3 (25/10/2018 to 04/11/20018): −0.15 °C on average; I4 (30/09/2018 to 14/10/2018): 6.56 °C on average; I5 (15/05/2019 to 22/05/2019): 13.22 °C on average. (**a**) E5 RSSI CDFs for GW1. (**b**) E5 RSSI CDFs for GW2. (**c**) E5 RSSI CDFs for GW4.

Our second target is to compare the RSSI by CloudRF to our measured RSSI data in order to observe how different distances and SF affect our previous conclusions. For that, we present now the results for the sensor E5, which is approximately 200 m away from E1. Figure 11 shows the RSSI CDFs for E5 sensor, similarly to Figure 10 for E1. For simplicity, we summarize the main conclusions for E5 in Table 9.

Comparing Figure 10 and Figure 11, we see lower RSSI values for E5 even it being 200 m closer than E1 for all gateways. The main reason is the higher SF of E5, providing superior RSSI reduction of the path gain by being closer. The RSSI reduction for higher SFs is also evaluated in Reference [47]. The conclusion about propagation models for E5 corroborates the findings for E1. In general, the Okumura-Hata model fits our data better than ITM but they are poor at capturing the weather seasonality, as expected.

**Figure 12:**
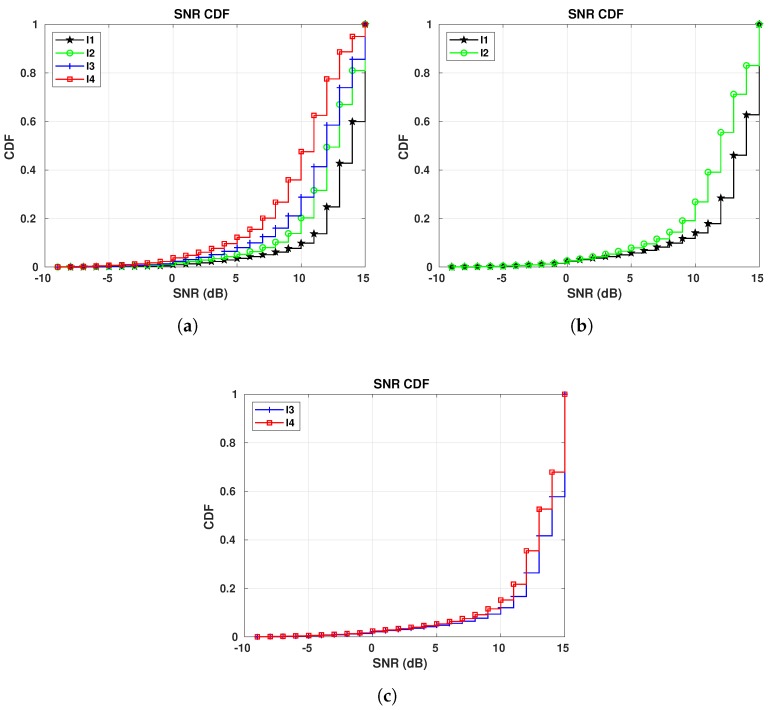
E1 SNR CDFs for all gateways in S1: I1 (16/01/2019 to 07/02/2019): −16.96 °C on average; I2 (22/12/2018 to 06/01/2019): −9.45 °C on average; I3 (25/10/2018 to 04/11/20018): −0.15 °C on average; I4 (30/09/2018 to 14/10/2018): 6.56 °C on average. (**a**) E1 SNR CDFs for GW1. (**b**) E1 SNR CDFs for GW2. (**c**) E1 SNR CDFs for GW4.

Our third target in S1 is to evaluate how the SNR is affected by changes in temperature. Figure 12 show the SNR CDFs for E1, each plot showing the values collected at one of our gateways. We present a single plot per gateway for all periods, in order to make the comparison process as easy as possible. When looking at the figures, we first notice that, as the temperatures start to drop, the SNR starts to increase. This is clear when we look at all plots—the curves appear in decreasing order, from the highest temperatures to the lowest ones (I4 to I1).

The effect of the temperature in the SNR, mainly in the calculation of the noise power, was first shown in Reference [48] and it is still used in recent literature [49]. The Johnson noise (or thermal noise) mentioned by Friis in Reference [48] is the one responsible for this phenomenon—when it is cold, the electrons move slowly, lowering the thermal noise, thus increasing the SNR and when it is warm, the electrons move faster, increasing the thermal noise, thus reducing the SNR. LoRa is especially susceptible to this effect, as it operates below the noise floor.

**Figure 13:**
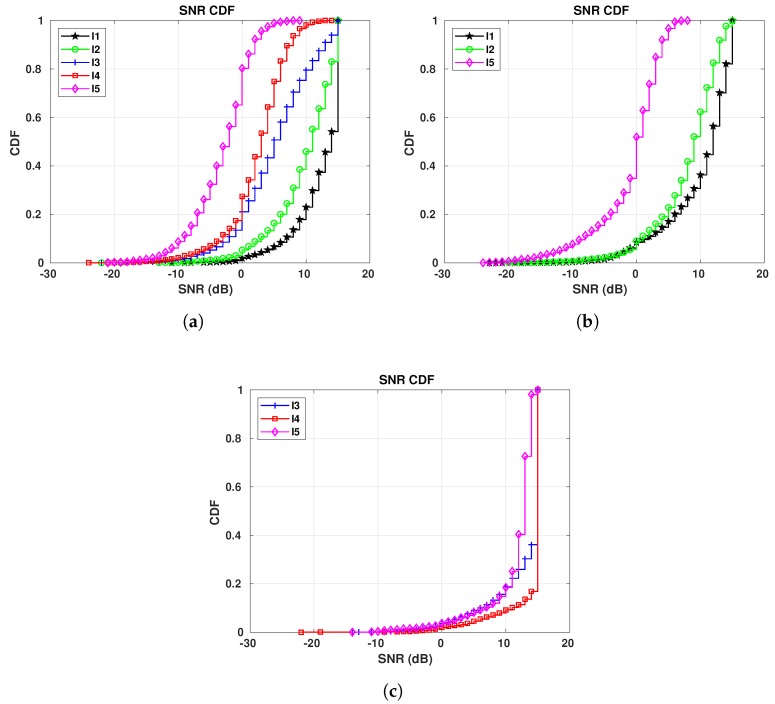
E5 SNR CDFs for all gateways in S1: I1 (16/01/2019 to 07/02/2019): −16.96 °C on average; I2 (22/12/2018 to 06/01/2019): −9.45 °C on average; I3 (25/10/2018 to 04/11/20018): −0.15 °C on average; I4 (30/09/2018 to 14/10/2018): 6.56 °C on average; I5 (15/05/2019 to 22/05/2019): 13.22 °C on average. (**a**) E5 SNR CDFs for GW1. (**b**) E5 SNR CDFs for GW2. (**c**) E5 SNR CDFs for GW4.

Figure 13 shows the SNR CDFs for E5 during different periods. They follow the same pattern of the SNR CDFs shown for E1—as lower is the temperature, as higher is the SNR. Taking a closer look at the results for GW4, shown in Figure 13c, we see that the curves for I3 and I5 are crossing each other, up to the 23rd-percentile. For those two periods, there are common temperatures during the days, that is, there are some days in I3 in which the maximum temperature was the same as the minimum temperature registered in some days during I5, even though the difference between the average temperatures for I3 and I5 is 13.37 °C. These weather conditions are responsible for the two curves of those different periods crossing each other.

So far, we have presented RSSI and SNR individually. However, we wanted to investigate if the weather had any impact in the joint relation of those two metrics. We decided to show this relation only for E5 at the gateway GW1, as the results for the other gateways and E1 sensor are similar.

It is worth mentioning that Kerlink’s network server allows us to extract two different values of RSSI, named RSSIC and RSSIS, the first representing the power at receiving antenna and the former measuring the power after LoRa processing.

Both values of RSSI depend on the SNR value. According to Kerlink’s documentation (which follows what is described in Reference [50]), RSSIS and RSSIC are defined as follow:

- If SNR ≥ 0: RSSIS = RSSIC = RSSI
- If SNR < 0: RSSIS = RSSIC + SNR

**Figure 14:**
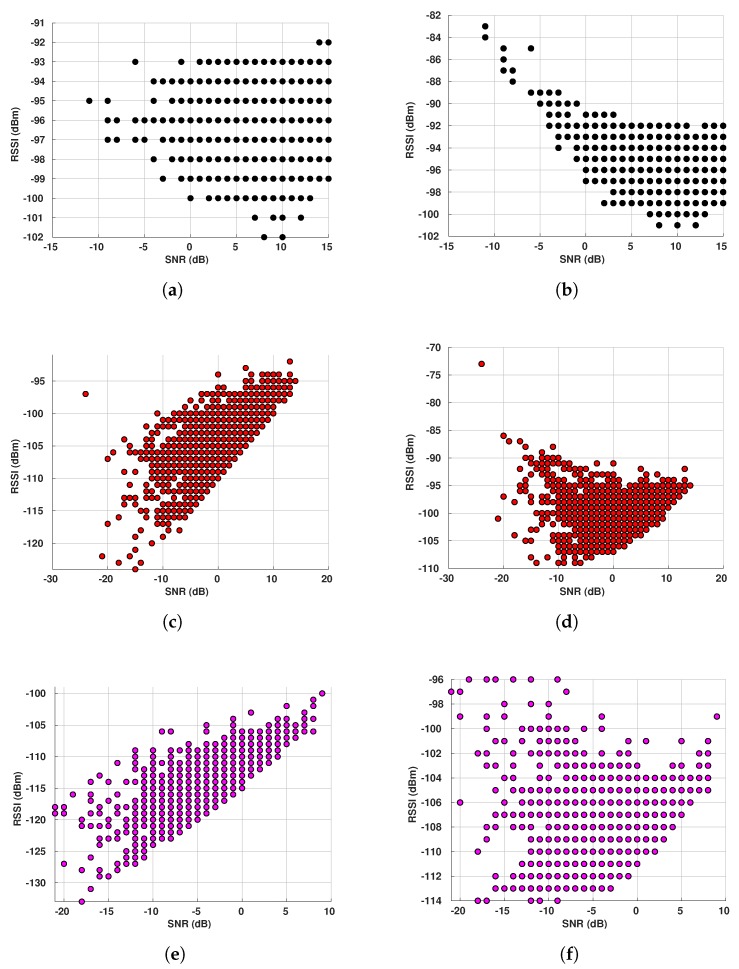
E5 RSSI x SNR for GW1 during different periods: I1 (16/01/2019 to 07/02/2019): −16.96 °C on average; I4 (30/09/2018 to 14/10/2018): 6.56 °C on average; I5 (15/05/2019 to 22/05/2019): 13.22 °C on average. (**a**) RSSI Signal—I1. (**b**) RSSI Channel—I1. (**c**) RSSI Signal—I4. (**d**) RSSI Channel—I4. (**e**) RSSI Signal—I5. (**f**) RSSI Channel—I5.

Figure 14 shows RSSI versus SNR for both RSSIs during different periods. The first we can notice is the decreasing pattern shown for RSSIC when the SNR has negative values, mainly during I1 and I4 periods. We can also see how the pattern changes when the SNR gets positive—the SNR increases as the RSSI increases. This is the expected behavior according to the RSSIC definition. Evaluating the RSSIS in Figure 14a,c,e, we reach two main conclusions—(i) the increasing pattern happens during I4 and I5, which shows the effect of SNR addition to the RSSI when the signal is below the noise floor, indicating that the channel is experiencing noise; and (ii) the increasing pattern does not happen during the coldest period (I1), which means that most of the time the channel is considerably good, allowing the network to operate above the noise floor, thus excluding the need for the SNR addition into the RSSI. Also, the RSSIS values are the highest during I1 (the coldest period), as shown in Figure 14a and the lowest RSSI values are observed during the warmest period (I5), as shown in Figure 14e. Finally, the shapes of our results become steeper as the temperature increases, as expected.

An important observation during our experiments is regarding the maximum SNR. From our results, the maximum SNR reported in all scenarios was 15 dB, which is an upper limit established by Semtech.

### 7.2. S2 Results

S2 is the scenario where we have an indoor sensor, which is 10 km away from the only gateway it reaches (GW1). As this sensor is operational since 20 October 2018, we only have data for I1, I2, I3 and I5 periods. It also has the ADR feature disabled both in the network server and itself.

**Figure 15:**
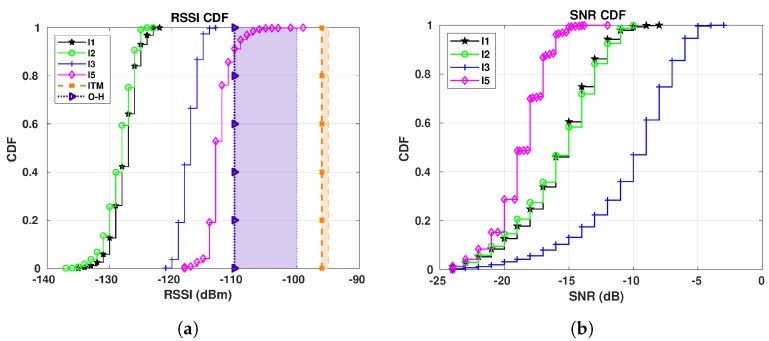
E8 CDFs for GW1 (RSSI and SNR): I1 (16/01/2019 to 07/02/2019): −16.96 °C on average; I2 (22/12/2018 to 06/01/2019): −9.45 °C on average; I3 (25/10/2018 to 04/11/20018): −0.15 °C on average; I5 (15/05/2019 to 22/05/2019): 13.22 °C on average. (**a**) E8 RSSI CDFs for GW1. (**b**) E8 SNR CDFs for GW1.

Figure 15 shows the RSSI and SNR CDFs for sensor E8.

As previously observed, the ITM range calculated by CloudRF (between −95 and −96 dBm) has overestimated the RSSI values, when compared to the values from our LoRaWAN and the Okumura-Hata model. All the curves of Figure 15a are to the left of both the range and the value calculated with the Best Server Feature and none of the curves touches the aforementioned values regardless of the propagation model. The only exception is for I5 period whose range by Okumura-Hata includes 14% of its values (from the 86th to the 100th-percentile).

The influence of weather in SNR for S2 is presented in Figure 15b. Different from S1, we have two extra constraints for S2 scenario—the distance between the gateway and the E8 sensor is approximately 10 km in a straight line and the fact that the sensor is indoor.

Comparing the results of Figure 15b with the SNR values shown in Section 6.1, the SNRs presented here are considerably lower than the ones observed for E1 and E5. As the signal strength falls off with distance [51] and the distance between E8 and GW1 is approximately 10 km in a straight line, this is expected, as with the drop in the signal also comes a drop in the SNR.

**Figure 16:**
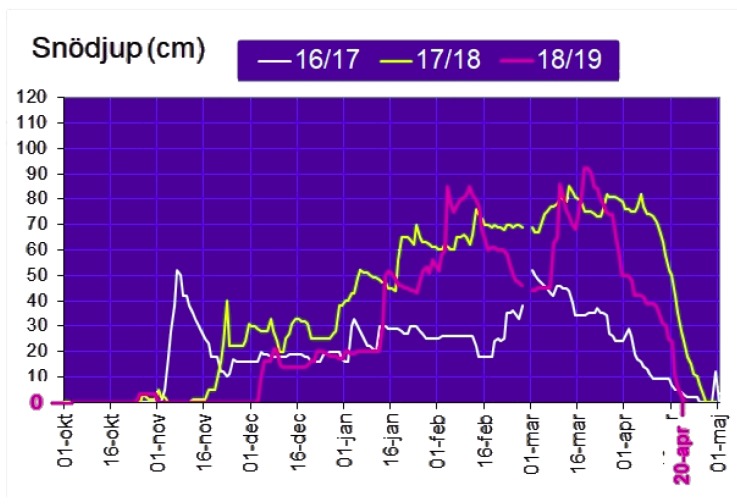
Snow Depth Chart for Skellefteå extracted from Reference [54]. The pink curve corresponds to the 2018/2019 period.

Although we expect a higher SNR with the cold, this is not the case in the results for E8. The best SNR values are the ones for I3 (25/10/2018–04/11/2018, with an average temperature of −0.15 °C), instead of the ones for I1 (16/01/2019–07/02/2019, with an average temperature of −16.96 °C). We believe this behavior is twofold—(i) the distance between E8 and GW1 (10 km) and (ii) the presence of snow in the environment [52,53] during I1 and I2. Figure 16 presents the snow depth chart [54], evidencing a considerable amount of snow in Skellefteå during such periods. The y-axis corresponds to the snow depth measured in centimeters (labeled as Snödjup) and the x-axis corresponds to the time of the year. The effect of the snow gets clear when we check the RSSI CDFs of Figure 15a—the RSSI curves for I1 and I2 have the lowest RSSI values among all the periods, meaning that the snow combined with long distance has a negative impact on both the RSSI and SNR. The effect of the snow has not been meaningful so far due to the small distance between the gateways and the sensors presented in Section 7.1.

**Figure 17:**
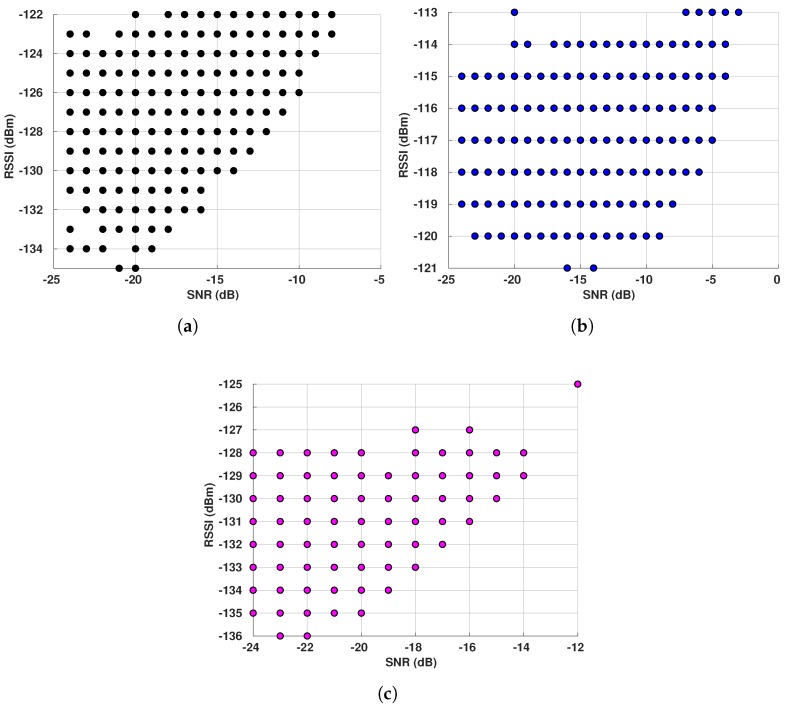
E8 RSSI versus SNR for GW1 during different periods: I1 (16/01/2019 to 07/02/2019): −16.96 °C on average; I2 (22/12/2018 to 06/01/2019): −9.45 °C on average; I3 (25/10/2018 to 04/11/20018): −0.15 °C on average; I4 (30/09/2018 to 14/10/2018): 6.56 °C on average; I5 (15/05/2019 to 22/05/2019): 13.22 °C on average. (**a**) I1 (−28.7 °C to −6.9 °C). (**b**) I3 (−11.4 °C to 7.8 °C). (**c**) I5 (3.2 °C to 24.8 °C).

Similar to Section 7.1, we show the RSSI Signal versus SNR for the E8 sensor in Figure 17. It is worth mentioning that from now on we will only present the RSSIS, as we intend to evaluate the behaviour of the signal after LoRa processing.

During all periods there is an increasing pattern—as the RSSI increases, the SNR also increases. Differently from results shown in Section 7.1, the I3 is the period with the highest RSSI values. As previously discussed, this is due to the snow during I1, which attenuates the signal, highlighting the linear relation between RSSI and SNR.

### 7.3. S3 Results

**Figure 18:**
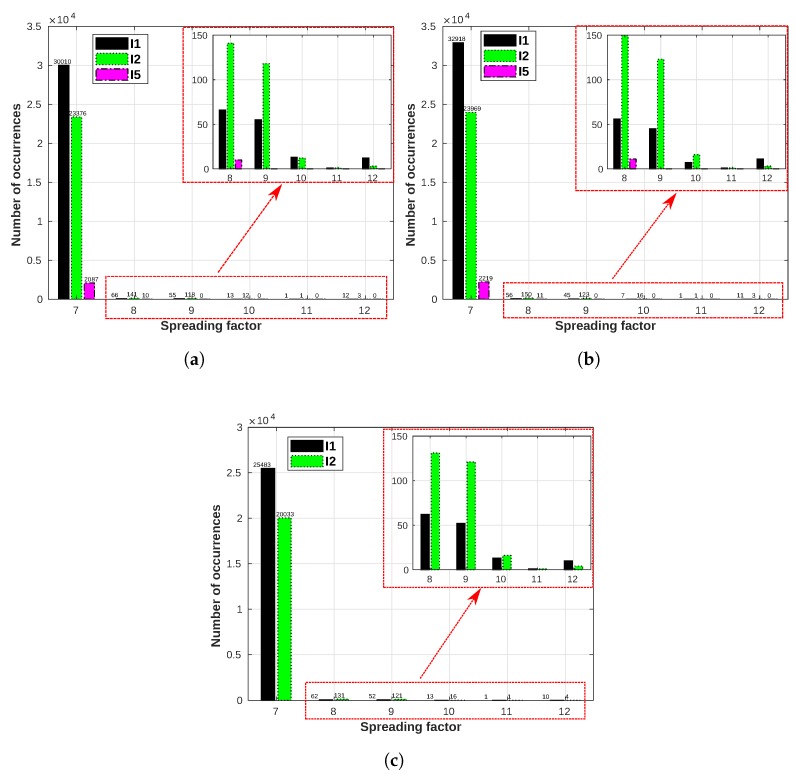
E6 SF usage for all gateways: I1 (16/01/2019 to 07/02/2019): −16.96 °C on average; I2 (22/12/2018 to 06/01/2019): −9.45 °C on average; I5 (15/05/2019 to 22/05/2019): 13.22 °C on average. (**a**) E6 SF usage for GW1. (**b**) E6 SF usage for GW2. (**c**) E6 SF usage for GW3.

The results for S3 show the usage of different SF when the devices are configured with ADR enabled. E6 and E7 are the devices used in this scenario. Figure 18 shows the SF usage for all gateways for E6. We present only for three gateways because E6 messages cannot reach GW4. The main reason is a small mountain (Klockarberget) between E6 and GW4, a mountain covered by a dense coniferous forest [55], causing signal blocking.

**Figure 19:**
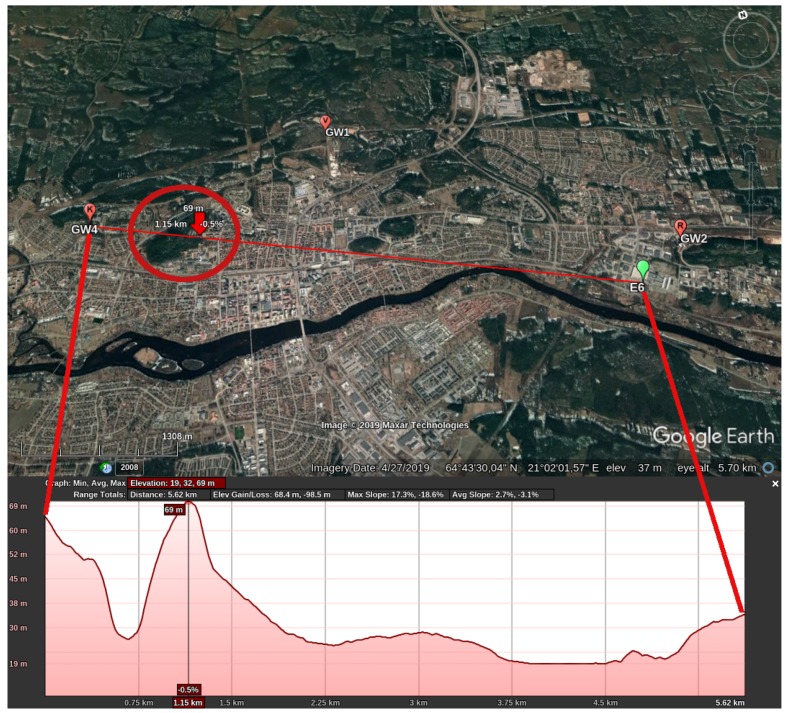
Elevation profile for the path between E6 and GW4 created with Google Earth [45].

According to Reference [49], the propagation loss calculation depends on the structure, density and type of trees, as well as the antenna height. In S3 scenario, E6 is located at the ground level, GW4 is at a height of 64 m and Klockarberget has a height of approximately 72 m, thus completely obstructing the LoS path between E6 and GW4. For verification purposes, the elevation profile for the path between E6 and GW4 is presented in Figure 19. The area in the figure inside the red circle corresponds to Klockarberget.

Another point to note for S3 is that according to Reference [3], in situations of constant changes in channel attenuation, ADR is not performed by the network server. Instead, the device’s application layer is responsible for the ADR and according to Reference [3], when this happens, the aggregated time on-air should be minimized. On the other hand, for situations of good propagation, ADR is performed by the network server, aiming at the most energy-efficient operation (for battery saving). Thus, ADR functionality tends to select the lowest SFs, which are the most energy-efficient and airtime-efficient. Results of Figure 18 confirm this behavior. For I1 and I2 periods, the channel between E6 and at least one of the gateways was reasonably good, which means that the network server select the most energy-efficient SFs. However, all the other SF are also used by E6 but considerably less often than the lowest ones. During I5 (the warmest period) only SFs 7 and 8 are selected by E6, although while SF7 is used 2087 times and 2219 times during I5 for GW1 and GW2, respectively, SF8 was selected 10 times and 11 times for GW1 and GW2. This clearly shows that—(i) the device is performing ADR when the channel changes in a stochastic fashion, as only the two lowest SF are selected and (ii) the SFs are selected to keep the time on-air as low as possible.

**Figure 20:**
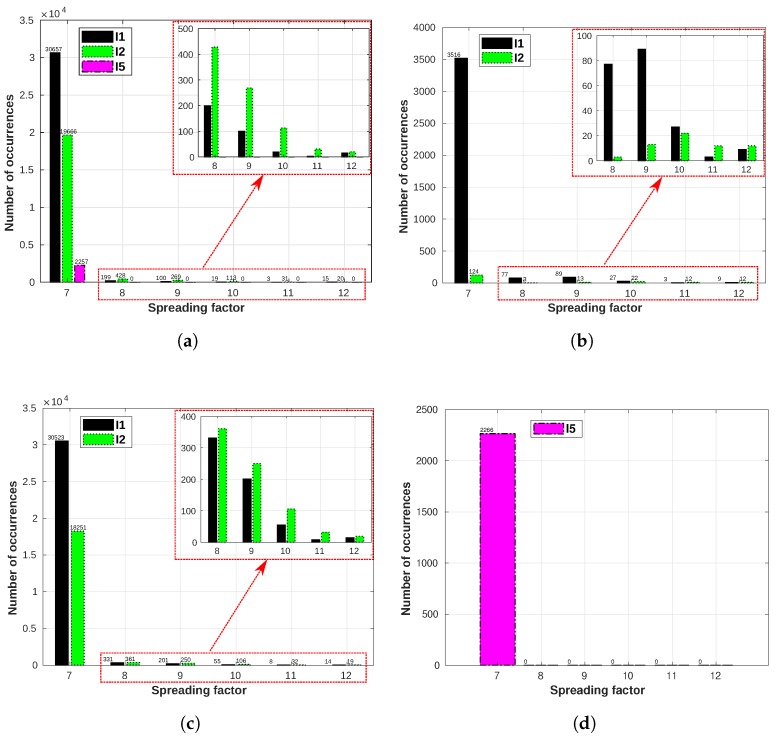
E7 SF usage for all gateways: I1 (16/01/2019 to 07/02/2019): −16.96 °C on average; I2 (22/12/2018 to 06/01/2019): −9.45 °C on average; I5 (15/05/2019 to 22/05/2019): 13.22 °C on average. (**a**) E7 SF usage for GW1. (**b**) E7 SF usage for GW2. (**c**) E7 SF usage for GW3. (**d**) E7 SF usage for GW4.

E7 is positioned in another part of the city (Figure 7) so that we would evaluate the channel conditions at a different location. Figure 20 shows the SF usage for all gateways for E7. When comparing the three periods, the last interval (I5) has a considerably lower number of occurrences than the intervals I3 and I4. This variation in data is linked to the power availability from lampposts (as they are only powered when the lampposts are lit), since these sensors are not battery powered and they switch off when the lampposts are switched off. In the city of Skellefteå, I1 and I2 are the periods of the year in which we have the darkest days (those are periods of the year when we also have a considerable amount of snow, thus E6 and E7 are equipped with tilt sensors to detect when a lamppost falls, which might happen due to the snowplows hitting the lampposts), meaning that the lampposts will be turned on for a longer time than during I5 (early summer), where we can have up to 18 hours of sunlight. Thus, the lamppost would be turned on for shorter periods during I5, leading to fewer messages being transmitted by both sensors E6 (Figure 18) and E7 (Figure 20).

The behavior for E7 during the warmest period (I5) is the same as E6—the lower SFs tend to be used more times than the highest SFs, in favor of energy and airtime efficiencies.

### 7.4. S4 Results

**Figure 21:**
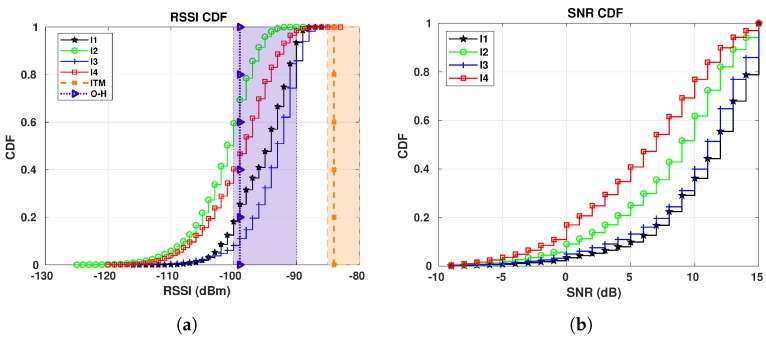
E1 RSSI and SNR CDFs for GW3: I1 (16/01/2019 to 07/02/2019): −16.96 °C on average; I2 (22/12/2018 to 06/01/2019): −9.45 °C on average; I3 (25/10/2018 to 04/11/20018): −0.15 °C on average; I4 (30/09/2018 to 14/10/2018): 6.56 °C on average. (**a**) RSSI CDFs. (**b**) SNR CDFs.

Figure 21 shows the RSSI and SNR CDF curves for E1 in relation to GW3. Figure 21a is in favor of Okumura-Hata model. Its estimation (between −90 and −100 dBm) shown in Figure 2e covers more than 60% of the values collected for I1, I3 and I4 periods, while for I2, a little less than 50% of the collected RSSI values fit the estimation. On the other side, ITM calculated range (−80 and −85 dBm) overestimates the RSSI values. This is clear when we look at the values calculated with the Best Server Feature—the vertical line for ITM crosses I3 and I4 curves at the 100th-percentile, meaning that just a few of our collected RSSI have the same value as the calculated one.

Analyzing Figure 21b the CDFs for I1 and I3 for GW3 are very close together, although the difference in temperature between those two periods is 16.81 °C on the average, which differs from the results for the other gateways. The reason is twofold—(i) the path between all sensors and this gateway is NLoS, causing higher signal variation; and (ii) the warm air expelled by the surrounding buildings and heated pathways are heating the area where GW3 is located, making the real temperature of the device and the environment temperature collected from Reference [42] unmatchable.

**Figure 22:**
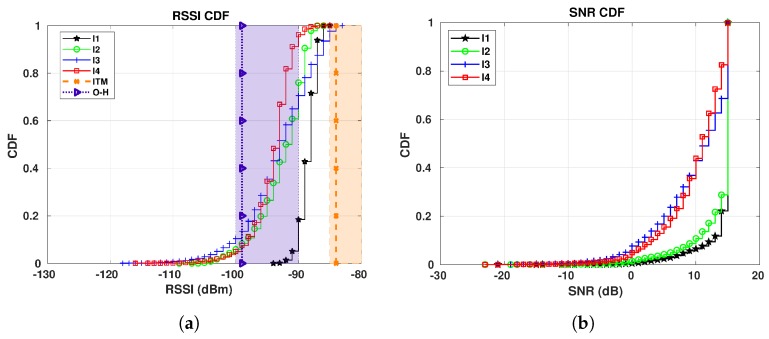
E5 RSSI and SNR CDFs for GW3: I1 (16/01/2019 to 07/02/2019): −16.96 °C on average; I2 (22/12/2018 to 06/01/2019): −9.45 °C on average; I3 (25/10/2018 to 04/11/20018): −0.15 °C on average; I4 (30/09/2018 to 14/10/2018): 6.56 °C on average. (**a**) RSSI CDFs. (**b**) SNR CDFs.

Figure 22 shows the results for E5. The conclusion about RSSI is the same as for E1—the Okumura-Hata is the model which best fits our data, while ITM overestimates the RSSI. Regarding the SNR, shown in Figure 22b, the SNRs for the colder and warmer periods are not that far apart. Again, this might be related to the heating from the surrounding buildings.

We omit the relation RSSI versus SNR for GW3 because it follows the same behavior as already shown for the other gateways.

## 8. Conclusions and Lessons Learned

We present an evaluation of the LoRaWAN assembled in the city of Skellefteå, in northern Sweden, in terms of RSSI, SNR and SF usage when ADR is enabled.

From the RSSI and SNR curves for the outdoor sensors, we can see that the results benefit from the cold. When the temperatures are low, the noise power is reduced, leading to better SNR values. However, we still need to consider the battery as a limiting factor in very cold weather, as the cold tends to drain the batteries quicker than when it is warm [56].

Our results also indicate a negative impact on LoRa performance with the combination of long distances and snow (Section 7.2). As Skellefteå is a region with a subarctic climate, it is covered in snow for at least 5 months per year. This must be taken into consideration when planning the positioning of both sensors and gateways, in a way to minimize the negative impact of snow in signal propagation. As further studies, we intend to evaluate how the snow influences the signal propagation considering different distances between the transmitter and the receiver.

The comparison between the RSSI values from our LoRaWAN with the results estimated by CloudRF shows that Okumura-Hata has better results when estimating the RSSI, while the ITM model tends to do an overestimation. Gateways GW1, GW3 and GW4 are the ones in which most of our RSSI values fit the Okumura-Hata model, while for GW2 both models had two or three curves fitting their ranges. We also see (from the curves in Figure 15a) the influence of the temperature in the RSSI values. Thus, there is a need to optimize some propagation models to consider the weather condition as an input parameter. As a future work, which is out of the scope of our research, we recommend the conception or evaluation of propagation models including the temperature as an input parameter (e.g., the attenuation by atmospheric gases model [57]), as well as the tuning of the Okumura-Hata model, which was the one presenting estimations closer to the results obtained from our LoRaWAN.

For the ADR evaluation performed in this work, we used a proprietary ADR model implemented at the network server (owned by Kerlink) and the application layer model implemented in devices E6 and E7. As further studies, we intend to implement our own ADR algorithm both on the network server and in the sensor, in order to compare with existing models and possibly enhance those models towards an energy-coverage-error tradeoff.

Two evaluations are left out of this work—the devices battery consumption and the packet delivery ratio. As the scope of this work is to characterize the channel in the smart region of Skellefteå at different periods of the year, the two aforementioned metrics are not considered. However, we intend to evaluate those parameters as part of future work.

As we perform measurements for a period of about eight months, we could share some lessons learned by our experience with LoRaWAN deployment. The first one is regarding the devices’ energy availability. As some of the devices we use are powered up by the lampposts where they are mounted (devices E6 and E7), we had fewer data during the early summer (I5 time period, 15/05/2019 to 22/05/2019), as during this time the lampposts would be turned on for very short times along a day. It would have been beneficial if we had different devices, possibly from other brands, powered by battery, instead of relying on the lamppost power. Secondly, when working with commercial tools (like the one used during our work for estimating the RSSI, CloudRF) it is important to consider that some features might be discontinued with upgrades, as it was the case for CloudRF. After an upgrade, CloudRF discontinued the ITWOM propagation model. Thirdly, the equipment position must be well planned, as it might not reflect the main objectives of the study. For example, we had surprising results from GW3, as it is positioned close to heat sources and it is in a NLoS position regarding the sensors used in our work. However, even if it was not our initial plan, it is worth as a realistic deployment scenario in an urban city.

The use of off-the-shelf solutions might limit the study due to absence of public information about the equipment. Thus, having access to the manufacturer of such devices is recommended (as it was our case). We exchanged several e-mails with the manufacturer of our gateways and network server (Kerlink) with questions regarding the gateways’ chipsets, the two RSSIs available in the network server and its difference (RSSIS and RSSIC) and we even asked about their ADR implementation, which they could not disclosure as it is a proprietary software. We then recommend researchers to use every kind of public information available, like the forums of most of the manufacturers, which are excellent sources of knowledge. Additionally, in long measurement campaigns (as it is the case presented in this study) it is important not to upgrade the equipment firmware, as some metrics can be reported at different manners for different software versions.

Generally speaking, our study indicates that one must take into account the environment temperature on the deployment plan of LoRaWAN, mainly because of its influence on the battery capabilities, the propagation conditions and the noise behavior. As temperature impacts on those factors, it also has a close relation to the expected coverage and transmission rates of LoRaWAN, becoming crucial for areas with high thermal amplitude.

## Figures and Tables

**Table 1:** LoRaWAN EU863-870 TX Data rate table (from Reference [31]).

Data Rate	Configuration	Indicative Physical Bit Rate (bits/s)
0	LoRa: SF12/125 kHz	250
1	LoRa: SF11/125 kHz	440
2	LoRa: SF10/125 kHz	980
3	LoRa: SF9/125 kHz	1760
4	LoRa: SF8/125 kHz	3125
5	LoRa: SF7/125 kHz	5470

**Table 2:** Gateways Location.

Gateway	Altitude (m)	Lat, Long
GW1	165	64.76736, 20.97684
GW2	76	64.74462, 21.04086
GW3	54	64.75089, 20.95882
GW4	66	64.76292, 20.92167

**Table 3:** Sensors Configuration and Location.

Sensor	Model	Measurements Capabilities	Spreading Factor	Reporting Interval	Coding Rate	Lat, Long
E1	Elsys ELT-1	Temperature, humidity, and orientation	7	1 min	4/5	64.748983, 20.912189
E2	Elsys ELT-1	Temperature, humidity, and orientation	10	5 min	4/5	64.749404, 20.912538
E3	Elsys ELT-1	Temperature, humidity, and orientation	10	3 min	4/5	64.750306, 20.914273
E4	Elsys ELT-1	Temperature, humidity, and orientation	12	3 min	4/5	64.749404, 20.912538
E5	Elsys ELT-1	Temperature, humidity, and orientation	12	5 min	4/5	64.750306, 20.914273
E6	mcf88 MCF-LW06485	Tilt (accelerometer), temperature, current, voltage, and sound	ADR	5 min	4/5	64.742171, 21.029322
E7	mcf88 MCF-LW06485	Tilt (accelerometer), temperature, current, voltage, and sound	ADR	5 min	4/5	64.764916, 20.904624
E8	Elsys ERS	Temperature, humidity, light, and motion	12	5 min	4/5	64.849365, 20.889126

**Table 4:** CloudRF common parameters for all scenarios.

Transmitter (Gateway)		Antenna (Gateway)		Receiver (Sensors)		CloudRF Output	
Frequency	868 MHz	Type	OEM Half-Wave Dipole	Height(s) AGL	2 m	Terrain resolution	10 m/ 33 ft
		Polarization	Vertical	Antenna Gain	3 dBi		
Gateway RF Power	20 dBm	Direction	0	Antenna Gain	3 dBi	Colour scheme	Greyscale GIS
		Tilt	0	Units of measurement	Received Power (dBm)		
Lat, long and height	From Table 2	Tx Gain	3 dBi	Units of measurement	Received Power (dBm)	Radius	10 km
		Feeder loss ${}^{1}$	0 dB 1 dB	Sensitivity	−140 dBm		

${}^{1}$ 0 dB for GW1 and GW4 and 1 dB for GW2 and GW3. The antennas of GW1 and GW4 are connected straight to the gateway, while the antennas of GW2 and GW3 are connected to the gateway via a 1 m long cable.

**Table 5:** CloudRF propagation models parameters.

Model			
ITM		Okumura-Hata (O-H)	
Reliability	90.00%	Environment	All gateways: Average
Terrain conductivity	GW1: Mountain/Sand	Knife-edge diffraction	off
	GW2 to GW4: City		
Radio climate	Maritime temperate (land)	Random clutter	0 m
Random clutter	0 m	Point clutter	off
Point clutter	off		

**Table 6:** RSSI values calculated with CloudRF Best Server Feature.

Propagation Model					
ITM			Okumura-Hata		
Sensor	Gateway	RSSI(dBm)	Sensor	Gateway	RSSI(dBm)
E1	GW1	−88	E1	GW1	−97
	GW2	−94		GW2	−111
	GW3	−84		GW3	−99
	GW4	−81		GW4	−92
E5	GW1	−87	E5	GW1	−96
	GW2	−94		GW2	−111
	GW3	−84		GW3	−99
	GW4	−80		GW4	−91
E8	GW1	−96	E8	GW1	−110

**Table 7:** Periods and temperature average of our evaluations.

Label	Temperature Average (°C)	Minimum Temperature (°C)	Maximum Temperature (°C)	Time Period	Equipment in Operation
I1	−16.96	−28.7	−6.9	16/01/2019–07/02/2019	GW1, GW2 and GW3 E1 to E8
I2	−9.45	−17.8	4.5	22/12/2018–06/01/2019	GW1, GW2 and GW3 E1 to E8
I3	−0.15	−11.4	7.8	25/10/2018–04/11/2018	GW1, GW3 and GW4 E1 to E5; E8
I4	6.56	−3.5	20.8	30/09/2018–14/10/2018	GW1, GW3 and GW4 E1 to E5
I5	13.22	3.2	24.8	15/05/2019–22/05/2019	GW1, GW2 and GW4 E2 to E8

**Table 8:** Measurement Scenarios (gateways, sensors, periods, metrics and assessment targets).

Scenario	Gateways/ Sensors	Measurement Periods	Evaluation Metrics	Assessment Targets
S1	GW1, GW2, GW4 E1 to E5	I1 to I5 ${}^{1}$	RSSI and SNR	Measured RSSI CDF vs CloudRF estimations per period Comparison of SNR CDFs per period RSSI vs SNR per period
S2	GW1, GW2, GW4 E8	I1, I2, I3, and I5	RSSI and SNR	Measured RSSI CDF vs CloudRF estimations per period Comparison of SNR CDFs per period RSSI vs SNR per period
S3	GW1, GW2, GW4 E6 and E7	I1, I2 and I5	SF histogram	ADR behavior per period
S4	GW3 E1 to E5	I1 to I4	RSSI and SNR SF histogram	Measured RSSI CDF vs CloudRF estimations per period Comparison of SNR CDFs per period

${}^{1}$ For E1 sensor, we only have data from I1 to I4, because E1’s battery had run out on 24 January 2019.

**Table 9:** Summary of the main conclusions for E5 in S1 (values from Figure 11).

Gateway	Map Range Estimation (dBm)	Best Server Feature (BSF) Estimation (dBm)	Main Conclusions
GW1	O-H: −100 to −90 ITM: −90 to −85	O-H: −95 ITM: −87	O-H: less than 50% of data is within the estimated range ITM: CDF curves are out of the estimated range ITM: estimation by the BSF does not cross any CDF ITM overestimated the RSSI values
GW2	O-H: −120 to −110 ITM: −95 to −90	O-H: −111 ITM: −94	O-H: very small parts of I2 is within the range O-H: 65% of I5 is within the range Neither ITM nor H-O fits I1 ITM overestimated the RSSI values
GW4	O-H: −100 to −90 ITM: −85 to −80	O-H: −91 ITM: −80	O-H: most of the data for I5 is within the range O-H: small parts of I3 and I4 are within the range O-H: estimation by the BSF crosses all the CDFs ITM: estimation by the BSF does not cross any CDF ITM: only parts of I3, I4 and I5 are within the range ITM overestimated the RSSI values

## Abbreviations

The following abbreviations are used in this manuscript:

**ADR**	Adaptive Data Rate
**API**	Application Programming Interface
**BSF**	Best Server Feature
**BW**	Bandwidth
**CDF**	Cumulative Distribution Function
**CR**	Coding Rate
**CSS**	chirp spread spectrum
**DR**	Data Rate
**IoT**	Internet of Things
**ISM**	Industrial, Scientific and Medical
**ITM**	Irregular Terrain Model
**ITWOM**	Irregular Terrain with Obstructions Model
**LoS**	Line-of-sight
**LoRaWAN**	LoRa Wide Area Network
**LPWA**	Low-Power Wide-Area
**MAC**	Medium Access Control
**NLoS**	Non-line-of-sight
**NTIA**	National Telecommunications and Information Administration
**PHY**	Physical
**REST**	Representational State Transfer
**RF**	Radio Frequency
**RSSI**	Received Signal Strength Indication
**RSSIC**	RSSI Channel
**RSSIS**	RSSI Signal
**RTE**	Radiative Transfer Engine
**SF**	Spreading Factor
**SNR**	Signal-to-Noise Ratio
**SSiO**	Societal development through Secure IoT and Open Data
